# Internal iteration gradient estimator based parameter identification for nonlinear sandwich system subject to quantized sensor and friction nonlinearity

**DOI:** 10.1371/journal.pone.0321175

**Published:** 2025-04-29

**Authors:** Huijie Lei, Yanwei Zhang, Xikun Lu

**Affiliations:** 1 School of Electronic, Electrical and Unmanned Aerial Vehicle, Anyang University of Technology, Anyang, People’s Republic of China; 2 Henan Angang Zhoukou Co., Ltd., Anyang, People’s Republic of China; Air University, PAKISTAN

## Abstract

This study proposes an internal iteration scalar-innovation gradient estimation method based on multi-innovation theory for a nonlinear sandwich system subject to a quantized sensor and friction nonlinearity. Different from the existing multi-innovation gradient method (MISG ), the proposed method is designed to resolve the existing shortages of the conventional MISG. First, the decomposing method is applied to derive the identification model, and the redundant parameter estimation problem is avoided. Then, an adaptive filter based on the prior knowledge of the system is proposed to obtain the helpful identification data. Second, to solve the multi-innovation length problem in MISG, the internal iteration principle is presented to convert the multi-innovation updating to scalar-innovation updating under a given innovation length, where the positive estimation performance can be achieved. Subsequently, the trigger mechanism is used to produce the suboptimal initial estimate value when the next parameter adaptive law is updated. Then, the fast convergence rate is obtained. Finally, the proposed estimation strategy is verified by conducting a numerical example and an experiment on a practical electromechanical system test bench.

## 1 Introduction

Ongoing progress in the development of affordable and smaller sensors and communication devices has driven the implementation of sensor networks in a variety of domains including traffic systems [1], military systems [2], wireless communication systems [3], and automatic control systems [4], etc. When a sensor system is developed, the inherent constraints must be considered, such as communication limitation, bandwidth, and sensor cost, because these factors affect system dynamic performance. One natural way is to compress sensor information using a quantizer to break through the inherent constraints. Thus, the control and system identification of systems with quantized data have sparked intense research interest in recent years.

As mentioned earlier, to save communication resources and overcome internal limitations, the collected data is often difficult to obtain with precision information, instead belongs to certain sets of data based on quantized sensor. Therefore, system identification based on quantised data better meets the demands of real-world system applications [5,6]. Leong [7] proposed a adaptive estimator to achieve the parameter recovery of FIR systems with quantized sensor. Wang [8] developed a recursive projection scheme for linear systems with quantized data. Guo [9] used a quasi-convex combination estimator for estimating the quantized systems. Given the intrinsic complexity and diversity of practical systems, using a uniform nonlinear model architecture to represent nonlinear systems can be difficult. In terms of the components of the system, one simple way to respect system modeling is to model the actual systems using a combination of linear systems and nonlinear submodels (i.e., block-oriented model) [10,11]. The block-oriented model can describe the linear dynamic as well as the nonlinear feature of systems, thus being easy to use and interpret. Thus, the modeling and identification of block-oriented models with a quantized sensor have become a significant area of interest in systems with quantized data. Early quantized block-oriented system identifications mainly focused on how to design input signals. For instance, Figwer [12] designed a multi-sinusoid excitation signal for Hammerstein system identification under quantized data to excite the modal information of the system. Furthermore, Zhao et al. [13] proposed an input signal with strongly scaled full-rank conditions for identifying a quantized Hammerstein system. In [14], Guo proposed a general input to achieve the parameter estimation of quantized systems. Subsequently, researchers shifted their focus to the design of identification algorithms. Guo [15] used a three-step scheme to estimate the parameters of Wiener-Hammerstein systems (i.e., sandwich systems) in a quantized environment. Li [16] introduced a robust adaptive method for resolving the parameter estimation problem of Wiener systems with binary-value data. Ding [6] studied the quantized Hammerstein estimation problem by proposing a kernel function optimization method. Zhao [4] presented an adaptive method for a block-oriented model system with quantized observations, and used a quantized block-oriented model to simulate the automotive engine system. In addition, time delay is also an important factor affecting the inherent constraints of the sensor networks [17–19]. Doostmohammadian [17] proposed a distributed estimation method to achieve the state estimation of linear networks with time-delay. Xu [20] presented an improved stochastic gradient descent algorithm for solving the parameter estimation of nonlinear exponential autoregressive time-series model. Chen [21] used a second-order optimization methods to estimate the parameter of nonlinear systems under time delay. The aforementioned identification methods are introduced by improving the performance of traditional algorithms to accommodate the identification of quantitative and time-delay systems.

With the rapid advancement of various applications in new fields, including sensor and control technologies [22], developing and studying novel identification algorithms based on new theories and methods are essential. During the past several decades, new theories and principles have been proposed and used to ameliorate the identification algorithm. For instance, Wang [23] combined multi-innovation theory to develop a recursive identifier to identify nonlinear systems. Zhou [24] proposed a gradient optimization algorithm based on a partially-coupled idea for a multivariate hybrid model. Chaudhary [25] proposed an improved gradient descent method by using the hierarchical identification idea for nonlinear systems. In addition, Ding [43] created a recursive generalized gradient estimator for Box-Jenkins systems by considering the principle of auxiliary model identification. Meanwhile, Hu [26] developed a fractional-order least squares method for a nonlinear system by force of fractional-order theory. Among the abovementioned estimators, algorithms that apply multi-innovation theory are popular estimation approaches. Many studies have been reported, such as those that use the multi-innovation gradient method [27], multi-innovation least squares method [28], multi-innovation norm method [29], and multi-innovation filter [30], etc. Although the multi-innovation algorithms can effectively identify the parameters of nonlinear systems, several inherent problems need further improvement.

As described earlier, the multi-innovation algorithm has two major shortcomings: the multi-innovation length problem and the initial value optimization problem. According to the theory of multi-innovation, the performance of identifier should continuously improve as the length of multi-innovation extends. However, many studies have shown that the estimation performance first rises and then falls as the length of multi-innovation increases. This peculiar phenomenon is mainly caused by the batch addition of noise data. The conventional approach is to use filters to address the system data. Even if the introduction of filters can reduce the effect of noise, noise and useful information in the system still coexist. Hence, the problem is not effectively solved. For the initial value optimization problem, one of several solutions is to use multistage identification schemes to obtain good initial values. However, multistage algorithms are highly computationally complex, and the estimation performance depends on the optimized value of the first step [15,31,32]. Based on the above analysis and motivation, this work introduces an efficient filter and auxiliary internal iteration mechanisms to solve the multi-innovation problem. Moreover, it considers the triggering mechanism to resolve the initial value problem.

As is well known, one reason why block-oriented model has unique advantages thanks to the existence of nonlinear submodels. Most practical mechanical systems usually consist of multiple components, thus inevitably forming a discontinuous system with contact interfaces [33]. When the system works, these contact interfaces will produce friction force [34,35]. Thus, friction is a common nonlinear characteristic that is widely present in various real-world systems. Under normal circumstances, the friction parameters are unknown, it is necessary to identify the friction parameters [36,37]. Pérez-Pérez [38] applied combined artificial neural network for obtaining the parameter information of friction model. Liang [39] proposed a separable least squares to identify the improved Stribeck friction model based on the compensation method. Feng [35] used a particle swarm optimization algorithm to estimate the friction model parameters accurately. Since the actual system can be composed of a combination of linear systems and nonlinear submodels, the identifications of block-oriented models with friction model have been studied. Assadi [40] proposed a Hammerstein with coulomb friction model to establish the dynamics of the electronic throttle body, and used the least squares method to identify the friction model. Li [41] studied the friction parameter estimations of Wiener-Hammerstein system with friction model by using an adaptive estimator. The above-mentioned friction models use either dynamic or static friction models to describe the characteristics of frictional forces. The models are designed for specific frictional characteristics and cannot describe most common frictional properties, making them generally less versatile.

**Fig 1:**
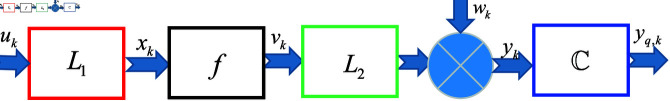
Quantized sandwich system.

Based on previous analysis, this study introduces an auxiliary internal iteration gradient optimization algorithm to resolve the parameter estimation of a nonlinear sandwich system under quantized observations, as displayed in Fig 1. First, a filter with a simplified structure is introduced to gain helpful quantized information from the collected data, which effectively alleviates the problem of estimation bias. Second, the auxiliary internal iteration principle is proposed to decompose the multi-innovation length with a batch update into scalar innovation with an internal iteration update, which addresses the multi-innovation length issue. To handle the initial value problem, the triggering mechanism is exploited to obtain the suboptimal estimates for each final value of the scalar innovation update, which avoids the heavy computational burden of multistage algorithms. In addition, the behavior of estimation error convergence is subjected to a thorough and rigorous analysis. Finally, the utility and efficacy of the proposed estimator are substantiated by an illustrative example and a real-world plant.

The contributions of this paper are enumerated as follows:

1) An internal iteration scalar innovation update is introduced into the multi-innovation estimator. Compared with the classic MISG [23,26], the developed method can avoid the batch addition of noise data, and the estimation performance is improved.
2) Devoted to combing the advantages of the triggering mechanism, the suboptimal selection method for the initial values of the parameter vector is integrated, so that a fast convergence rate is obtained by reducing the search time for real values [15,31,32].
3) Compared with the existing filter, the developed filter can extract more helpful quantized data because such filter takes into account the prior knowledge of the quantized system data [42,43].
4) The convergence of the proposed estimator is analyzed by introducing a Lyapunov-like function, thus demonstrating that the convergence error can converge to zero.

The rest of the report is listed as follows. Sect 2 introduces the identification problem with quantized data constraint. Sect 3 describes the proposed identifier with a novel adaptive law. In Sect 4, the estimator performance of the developed algorithm is discussed. In Sect 5, some statistical results are shown for validating the efficiency of the main findings. Sect 6 provides this paper and lists future potential work.

## 2 Problem statement

Consider the nonlinear Sandwich systems under binary-value sensor as


$$\begin{array}{ccc} L_{1}:x_{k}=\frac{A(z^{-1})}{B(z^{-1})}u_{k}, & & \end{array}$$ (1)

f:vk=γ1xk−γ1xk−1+α1sign(xk−xk−1)−α2 tanh⁡(xk−xk−1), (2)

$$\begin{array}{ccc} L_{2}:y_{k}=\frac{C(z^{-1})}{D(z^{-1})}v_{k}+\frac{w_{k}}{D(z^{-1})}, & & \end{array}$$ (3)

$$\begin{array}{ccc} y_{q,k}=C(y_{k}), & & \end{array}$$ (4)

$$\begin{array}{ccc} C(y_{k})=\{\begin{array}{cc} -1,\; & \;\text{when}\;y_{k}>1\\ 1,\; & \;\text{when}\;y_{k}<1.\\ \; \end{array} & & \end{array}$$ (5)

where $\frac{A(z^{-1})}{B(z^{-1})}$ and $\frac{C(z^{-1})}{D(z^{-1})}$ are transfer functions of $L_{1}$ and $L_{2}$, respectively. Their expressions are: $A(z^{-1})=\sum_{j=1}^{n_{a}}a_{j}z^{-i}$, $B(z^{-1})=1$ + $\sum_{i=1}^{n_{b}}b_{i}z^{-i}$, $C(z^{-1})=\sum_{j=1}^{n_{c}}c_{j}z^{-j}$, and $D(z^{-1})=1$  +  $\sum_{i=1}^{n_{d}}d_{i}z^{-i}$, and *z*^–1^ is backward shift operator, i.e., $z^{-1}u_{k}=u_{k-1}$. The system input and output signal are denoted by $u_{k}$, and $y_{k}$, separately. $w_{k}$ and $y_{q,k}$ are corresponding noise and quantized data. $C(y_{k})$ is binary senor.

**Fig 2:**
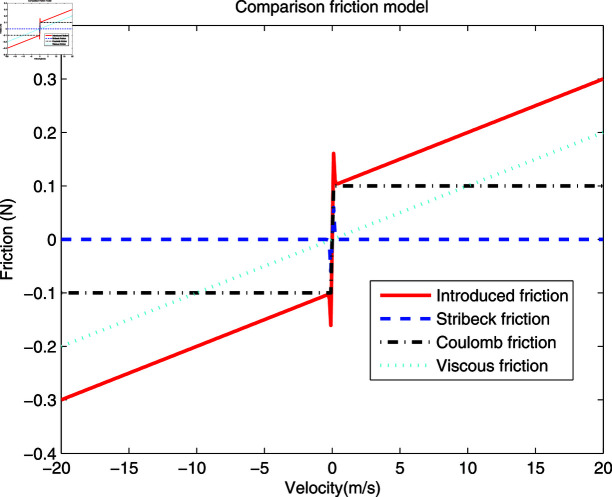
Friction model.

As a general rule, friction damages working surfaces, affects mechanical performance, increases energy consumption, and reduces the lifespan of components. To eliminate this impact, using nonlinear system identification techniques in practical control systems is often necessary to estimate the parameters of friction force and establish a nonlinear compensation [44,45]. As shown in Fig 2, the friction model of this paper can describe the features of common friction models such as Viscous friction, Coulomb friction and Stribeck friction [46], which is the motivation for introducing this model in this work.

In order to achieve the purpose of this paper, several common assumptions are introduced [47–49].

**Assumption 1**. *There exist no cancellation of zeros and poles in $\frac{A(z^{-1})}{B(z^{-1})}$ and $\frac{C(z^{-1})}{D(z^{-1})}$.*
**Assumption 2**. *The parameters $a_{1}=1$ and $c_{1}=1$ are set to one.*
**Assumption 3**. *The noise $w_{k}$ and the input signal $u_{k}$ are independent of each other.*
**Assumption 4**. *The orders $n_{a}$, $n_{b}$, $n_{c}$ and $n_{d}$ of the polynomial are given, and the coefficients $a_{j}$, $b_{i}$, $c_{j}$ and $d_{i}$ are unknown.*

Assumption 1 indicates that two linear dynamics are stable. In Assumption 2, the unique parameter model assumption is given [41,50]. Assumption 3 shows the work condition of noise data. Assumption 4 introduces the purpose of this paper.

By using the key item separation idea [42] and combining (1)–(3), we have


$$\begin{array}{ccc} y_{k}=\varphi_{k}^{T}\theta +w_{k}, & & \end{array}$$ (6)

where information vector $\varphi_{k}$ and parameter vector *θ* are defined as

$\varphi_{k}=[u_{k-2},u_{k-3},\cdots \;,u_{k-n_{a}-1},-x_{k-2},-x_{k-3},\cdots \;,-x_{k-n_{b}-1},sign(x_{k-1}$ − $x_{k-2}),-\tanh(x_{k-1}$  −  $x_{k-2}),v_{k-2},v_{k-3},\cdots \;,v_{k-n_{c}},-y_{k-1},-y_{k-2},\cdots \;,-y_{k-n_{d}}{]}^{T}$,

and


$$\begin{array}{cccc} \begin{array}{c} \theta =[c_{1}\gamma_{1}a_{1},c_{1}\gamma_{1}a_{2},\cdots \;,c_{1}\gamma_{1}a_{n_{a}},c_{1}\gamma_{1}b_{1}+c_{1}\gamma_{1},c_{1}\gamma_{1}b_{2},c_{1}\gamma_{1}b_{3},\cdots c_{1}\gamma_{1}b_{n_{b}},c_{1}\alpha_{1},c_{1}\alpha_{2},\\ \;\;c_{2},\cdots \;,c_{n_{c}},d_{1},d_{2},\cdots \;,d_{n_{d}}{]}^{T}. \end{array} & & & \end{array}$$

Considering the quantized sensor, the identification model is written as:


$$\begin{array}{ccc} \begin{array}{ccc} y_{k} & =\varphi_{k}^{T}\theta +w_{k}, & \\ y_{q,k} & =C(y_{k}). \end{array} & & \end{array}$$ (7)

The main goal of this paper is to present an improved multi-innovation estimator, to achieve the estimates of the parameter vector *θ* based on the acquisition data $\left\{u_{k},y_{q,k}\right\}$, and to validate the availability of the proposed identification algorithm by using some examples.

## 3 Internal iteration scalar-innovation gradient method

In current section, we will now describe how to apply the developed filter, internal iteration update idea and trigger mechanism to improve the estimation performance of the MISG algorithm.

Based on the expression of data vector and quantized output data (6)–(7), we known that $\varphi_{k}$ and $y_{q,k}$ contain the noise data, which should be filtered by using a filter.

Define $y_{f,k}$ and $\varphi_{f,k}$, we have


$$y_{f,k}=\frac{\eta_{k}}{\eta_{k}+1}y_{f,k-1}+\frac{1}{\eta_{k}+1}y_{q,k},$$ (8)

$$\varphi_{f,k}=\frac{\eta_{k}}{\eta_{k}+1}\varphi_{f,k-1}+\frac{1}{\eta_{k}+1}\varphi_{k},$$ (9)

$$\eta_{k}=\eta_{k-1}+\xi ∥\varphi_{k}-\varphi_{k-1}∥,$$ (10)

where $\eta_{k}$ is the proposed filter that is developed by using the quantized system data. The symbol  ∥ ⋅ ∥  is two-norm. *ξ* describes a learning rate.

**Remark 1**. *Unlike the existing filters, the presented filter (10) considers the prior knowledge of the system $\varphi_{k}$ − $\varphi_{k-1}$ to achieve the real-time adjustment of the filtering performance based on the changes in the quantized observation data.*

By using the multi-innovation identification theory [51], typical MISG algorithm are as follows:


$$\Xi_{\mu ,k}=Y_{\mu ,k}-\Phi_{\mu ,k}^{T}{\hat{\theta }}_{k-1},$$ (11)

$$R_{\mu ,k}=R_{\mu ,k}+∥\Phi_{\mu ,k}{∥}^{2},$$ (12)

$${\hat{\theta }}_{k}={\hat{\theta }}_{k-1}+\frac{\Phi_{\mu ,k}}{R_{\mu ,k}}\Xi_{\mu ,k},$$ (13)

where *μ* is multi-innovation length, $\Xi_{\mu ,k}$ is multi-innovation vector which is given as $\Xi_{\mu ,k}={\left[e_{k},e_{k-1},\cdots \;,e_{k-\mu +1}\right]}^{T}$, $\Phi_{\mu ,k}$ is multi-innovation quantized data matrix, it has $\Phi_{\mu ,k}=[\varphi_{k},\varphi_{k-1},\cdots \;,\varphi_{k-\mu +1}]$. $Y_{\mu ,k}$ represents multi-innovation quantized output vector, is has $Y_{\mu ,k}={\left[y_{q,k},y_{q,k-1},\cdots \;,y_{q,k-\mu +1}\right]}^{T}$. $R_{\mu ,k}$ is learning step.

Different from the conventional stochastic gradient optimization algorithm, MISG approach (11)–(13) can use quantized data at time *k*, *k*–1, ⋯ , *k* − *μ* + 1, thus achieving better identification performance [52]. However, when the multi-innovation vector $\Xi_{\mu ,k}$ (11) is applied to update the parameter vector ${\hat{\theta }}_{k}$ (13), the useful quantized system data and noise data are also used simultaneously, showing that as the multi-information length extends, the estimation performance initially enhances, subsequently diminishes. This phenomenon is known as the problem of multi-information length, which suppresses estimation performance. The primary cause of this phenomenon is that bulk noise data are continuously fed into the estimator because the system data are used for parameter updates. When the *μ* reaches a certain length, the utilization efficiency of bulk noise data increases, thus deteriorating its performance. Therefore, determining how to modify the utilization rate of bulk noise data is crucial for solving the above issue.

In order to realize this objective, we integrate the internal iteration idea into the MISG approach (11)–(13) to transform the utilization mode of bulk noise data. Define the internal iteration output vector $y_{k,j}\in \{y_{f,k},y_{f,k-1},\cdots \;,y_{f,k-\mu +1}\}$, and internal iteration data matrix $\varphi_{k,j}\in \{\varphi_{f,k},\varphi_{f,k-1},\cdots \;,\varphi_{f,k-\mu +1}\}$, the proposed algorithm is written as


$$\begin{array}{ccc} e_{k,j}=y_{f,k,j}-\varphi_{f,k,j}^{T}\theta_{k,j-1}, & & \end{array}$$ (14)

$$\begin{array}{ccc} R_{k,j}=R_{k,j-1}+∥\varphi_{f,k,j}{∥}^{2}, & & \end{array}$$ (15)

$$\begin{array}{ccc} {\hat{\theta }}_{k,j}={\hat{\theta }}_{k,j-1}+\frac{\varphi_{f,k,j}}{R_{k,j}}(y_{f,k,j}-\varphi_{f,k,j}^{T}\theta_{k,j-1}), & & \end{array}$$ (16)

where *j* ∈ { 1 , 2 , ⋯ , *μ* } , $e_{k,j}$ is scalar-innovation.

**Remark 2**. *From Eq (16), compared with the multi-innovation gradient method (11)–(13), the bulk updating of $\Xi_{\mu ,k}$ in (13) is transformed into the updating of the scalar-innovation data $e_{k,j}$ in (16) when parameter adaptive law is updated, reducing the amount of noise added and improving the multi-innovation length problem. When *j* = *j* + 1, the next scalar-innovation update is activated. By repeatedly iterating the aforementioned program, the parameter estimation is continuously updated until the end of the execution.*

Although the method (14)–(15) can enhance the estimation performance of the MISG approach, the initial value problem has not been solved. To solve the initial value problem, combining with the characteristics of internal update algorithm (14)–(15) and the trigger update mechanism [53], the following initial value optimization method is proposed as follow:


$$R_{k,0}=R_{k-1,\mu },$$ (17)

$${\hat{\theta }}_{k,0}={\hat{\theta }}_{k-1,\mu }.$$ (18)

**Remark 3**. *It can be observed from (17)–(18) that when *j* = *μ* at time *k* is finished, and the next innovation update mechanism is activated when *k* = *k* + 1. Then, the final values of time *k* (i.e, $R_{k,\mu }$, ${\hat{\theta }}_{k,\mu }$) are selected as the initial values of time *k* + 1 (i.e, $R_{k+1,0}$, ${\hat{\theta }}_{k+1,0}$). This process is equivalent to obtaining suboptimal initial values for parameter estimation updates and step size after each run, which reduces the algorithm’s optimization time for true values and accelerates the convergence speed of the algorithm. Compared with the multi-stage algorithms [54,55], the proposed method (17)–(18) persistently optimizes the initial values of parameter estimation updates, rather than the first optimization.*

It is noteworthy that as shown in Fig 1, the intermediate signals $x_{k}$ and $v_{k}$ cannot be directly measured. When the proposed method is conducted, these variables need be estimated. Numerous solutions address the aforementioned issue, one of the effective and widely utilized method is the auxiliary model approach. A substantial body of productive and excellent reports have been published [43,56,57]. The fundamental idea is to design auxiliary models for the corresponding unknown variables based on the raw system. By using the outputs of these auxiliary models to replace the unknown variables, these variables become indirectly measurable. According to the raw system (1)–(3), the auxiliary models $x_{ref,k}$ and $v_{ref,k}$ are constructed as


$$x_{ref,k}=\frac{Â(z^{-1})}{\hat{B}(z^{-1})}u_{k}$$ (19)

vref,k=γ^1xref,k−γ^1xref,k−1+α^1sign(xref,k−xref,k−1)−α^2 tanh⁡(xref,k−xref,k−1). (20)

In order to facilitate readers’ understanding of the proposed algorithm, the following flowchart of the estimator work is provided.

**Step (1)**: Set initial values: data length *N*, parameter vector ${\hat{\theta }}_{0,0}=I∕p0$, innovation length *μ*, and step $R_{0,0}=1$, adaptive filter $\eta_{0}$, learning rate *ξ*,
**Step (2)**: Gather the quantized data $\left\{u_{k},y_{q,k}\right\}$, calculate the information data $\varphi_{k}$ by (6),
**Step (3)**: Calculate the filtered vectors $y_{f,k}$ by (8), $\varphi_{f,k}$ by (9) with $\eta_{k}$ by (10),
**Step (4)**: Calculate the innovation $e_{k,j}$ by (14), $R_{k,j}$ by (15),
**Step (5)**: Update the estimated vector ${\hat{\theta }}_{k,j}$ by (16),
**Step (6)**: Calculate auxiliary model $x_{ref,k}$ and $v_{ref,k}$ by (19) and (20), respectively and use initial value optimization (17) and (18),
**Step (7)**: When *k* ≥ *N*, the estimation process ends, otherwise *k* : = *k* + 1, and skip to the step (2),
**Step (8)**: Obtain the final parameter estimate ${\hat{\theta }}_{N,\mu }$, end.

## 4 Convergence analysis

This section derives the convergence of the presented identification approach by means of the martingale sequence theory.

Let us suppose, the  {ℱ(k)} is established by using $w_{k}$, we named $\left\{w_{k},ℱ(k)\right\}$ as a bounded martingale sequence. Suppose the additive noise $w_{k}$ satisfies [58]:

(F1) $E[w_{k}|ℱ(k-1)]=0$,
(F2) $E[∥w_{k}{∥}^{2}|ℱ(k-1)]=\sigma_{w,t}^{2}\leq \sigma_{w}^{2}<\infty$,
(F3) $\underset{t\to \infty }{\mathrm{limsup}}\frac{1}{k}\overset{k}{\underset{g=1}{\sum }}∥w_{g}{∥}^{2}\leq \sigma_{w}^{2}<\infty$.

**Theorem 1**. *For the quantized sandwich systems (1)–(5) and the presented method (14)–(18), when $u_{k}$ is PE condition, then $R_{k}=O(\lambda_{min}[\overset{k}{\underset{m=1}{\sum }}\overset{\mu }{\underset{n=1}{\sum }}{\hat{\varphi }}_{f,m,n}{\hat{\varphi }}_{f,m,n}^{T}]),k\to \infty$, and (F1), (F2) and (F3) hold. Then, the estimates parameter error can converge to zero, that is, $∥{\hat{\theta }}_{k,\mu }$ − $\theta {∥}^{2}\to 0,k\to \infty$.*

Proof. Based on (16) with subtracting *θ*, it can be written as follows:


$$\begin{array}{cccc} {\tilde{\theta }}_{k,j} & ={\tilde{\theta }}_{k,j-1}+\frac{1}{R_{k,j}}{\hat{\varphi }}_{f,k,j}[y_{f,k,j}-{\hat{\varphi }}_{f,k,j}^{T}{\hat{\theta }}_{k,j-1}] & & \\ & ={\tilde{\theta }}_{k,j-1}+\frac{1}{R_{k,j}}{\hat{\varphi }}_{f,k,j}[-\tilde{y}(k,j)+w_{k,j}], \end{array}$$ (21)

where $\tilde{y}(k,j)={\hat{\varphi }}_{f,k,j}^{T}{\tilde{\theta }}_{k,j-1}+({\hat{\varphi }}_{f,k,j}^{T}-\varphi_{f,k,j}^{T})\theta_{k,j-1}$.

By using the definition of norm and combining (21), it yields:


$$\begin{array}{ccc} ∥{\tilde{\theta }}_{k,j}{∥}^{2}= & ∥{\tilde{\theta }}_{k,j-1}{∥}^{2}+\frac{∥{\hat{\varphi }}_{f,k,j}{∥}^{2}}{R_{k,j}^{2}}∥-\tilde{y}(k,j)+w_{k,j}{∥}^{2}+2\frac{{\tilde{\theta }}_{k,j-1}{\hat{\varphi }}_{f,k,j}^{T}}{R_{k,j}} & \\ & \times [-\tilde{y}(k,j)+w_{k,j}]\\ = & ∥{\tilde{\theta }}_{k,j-1}{∥}^{2}+\frac{-2R_{k,j}+∥{\hat{\varphi }}_{f,k,j}{∥}^{2}}{R_{k,j}^{2}}∥\tilde{y}(k,j){∥}^{2}+\frac{∥{\hat{\varphi }}_{f,k,j}{∥}^{2}∥w_{k,j}{∥}^{2}}{R_{k,j}^{2}}\\ & +\frac{2R_{k,j}-2∥{\hat{\varphi }}_{f,k,j}{∥}^{2}}{R_{k,j}^{2}}[\tilde{y}(k,j)w_{k,j}]. \end{array}$$ (22)

By using the scaling method to (22), we get:


$$\begin{array}{ccc} T_{\mu ,k}\leq & T_{\mu ,k-1}+\overset{\mu }{\underset{j=1}{\sum }}\frac{∥{\hat{\varphi }}_{f,k,j}{∥}^{2}∥w_{k,j}{∥}^{2}}{R_{k,j}^{2}}-\overset{\mu }{\underset{j=1}{\sum }}\frac{1}{R_{k,j}^{2}}∥\tilde{y}(k,j){∥}^{2} & \\ & +\frac{2R_{k,j-1}}{R_{k,j}^{2}}[\tilde{y}(k,j)w_{k,j}], \end{array}$$ (23)

where $T_{j,k}=∥{\tilde{\theta }}_{k,j}{∥}^{2}$.

Because


$$\overset{\mu }{\underset{j=1}{\sum }}\overset{\infty }{\underset{k=1}{\sum }}\frac{∥{\hat{\varphi }}_{f,k,j}{∥}^{2}}{R_{k,j}^{2}}\leq \overset{\mu }{\underset{j=1}{\sum }}\overset{\infty }{\underset{k=1}{\sum }}\frac{R_{k,j}-R_{k,j-1}}{R_{k,j-1}R_{k,j}}=\overset{\mu }{\underset{j=1}{\sum }}\left[\frac{1}{R_{0,j}}-\frac{1}{R_{\infty ,j}}\right]<\infty ,$$ (24)

we can see that (24) is bounded, and $\tilde{y}(k,j),{\hat{\varphi }}_{f,k,j}$, $R_{k,j}$ and $w_{k,j}$ are not related. By using the definition of the expectation and combining (F1)-(F3), (23) has the following expression:


$$E[T_{\mu ,k}|ℱ(k-1)]\leq T_{\mu ,k-1}+\frac{\mu \sigma_{w}^{2}}{R_{k,j}^{2}}\overset{\mu }{\underset{j=1}{\sum }}∥{\hat{\varphi }}_{f,k,j}{∥}^{2}-\overset{\mu }{\underset{j=1}{\sum }}\frac{1}{R_{k,j}}∥\tilde{y}(k,j){∥}^{2}.$$ (25)

Based on (24), the following inequality holds:


$$\frac{\mu \sigma_{w}^{2}}{R_{k,j}^{2}}\overset{\mu }{\underset{j=1}{\sum }}\overset{\infty }{\underset{k=1}{\sum }}∥{\hat{\varphi }}_{f,k,j}{∥}^{2}<\infty .$$ (26)

By applying martingale convergence theorem [58] to (25) and using (26), we have


$$E[∥{\tilde{\theta }}_{\mu ,k}{∥}^{2}]\to T_{0},k\to \infty ,$$ (27)

and


$$\underset{k\to \infty }{\lim}\overset{\mu }{\underset{j=1}{\sum }}\overset{k}{\underset{m=1}{\sum }}\frac{1}{R_{m,j}}∥\tilde{y}(m,j){∥}^{2}<\infty ,a.s.$$ (28)

where $T_{0}$ represents a random variable.

Based on (28), we known that when *k* → *∞*, $R_{k,j}\to \infty$ holds. By exploiting Kronecker Lemma [59] to (28), we have


$$\overset{\mu }{\underset{j=1}{\sum }}\frac{1}{R_{m,j}}\overset{k}{\underset{m=1}{\sum }}∥\tilde{y}(m,j){∥}^{2}\to 0,\;k\to \infty .$$ (29)

Because of $\tilde{y}(k,j)={\hat{\varphi }}_{f,k,j}^{T}{\tilde{\theta }}_{k,j-1}$, the following expression holds:


$$\tilde{y}(k,j)={\hat{\varphi }}_{f,k,j}^{T}({\tilde{\theta }}_{k,j}-\frac{1}{R_{k,j}}{\hat{\varphi }}_{f,k,j}(-\tilde{y}(k,j)+w_{k,j})).$$ (30)

By using the definition of the determinant, for (30), we obtain:


$$\begin{array}{cccc} ∥{\hat{\varphi }}_{f,k,j}^{T}{\tilde{\theta }}_{k,i-1}{∥}^{2} & \leq 2∥\tilde{y}(k,j){∥}^{2}+2∥{\hat{\varphi }}_{f,k,j}{∥}^{2}\overset{j-1}{\underset{i=0}{\sum }}\frac{∥\varphi_{f,k-i,j}{∥}^{2}}{∥R_{k-i,j}{∥}^{2}}∥\tilde{y}(k-i,j){∥}^{2} & & \\ & -4∥{\hat{\varphi }}_{f,k,j}{∥}^{2}∥\overset{j-1}{\underset{i=0}{\sum }}\frac{∥\varphi_{f,k-i,j}{∥}^{2}}{∥R_{k-i,j}{∥}^{2}}\tilde{y}(k-i,j)w_{k-i,j} & & \\ & +2∥{\hat{\varphi }}_{f,k,j}{∥}^{2}\overset{j-1}{\underset{i=0}{\sum }}\frac{∥\varphi_{f,k-i,j}{∥}^{2}}{∥R_{k-i,j}{∥}^{2}}∥w_{k-i,j}{∥}^{2} \end{array}$$ (31)

By using the scaling method to (31), it yields


$$\begin{array}{cccc} Z_{\mu ,k} & \leq 2\overset{\mu }{\underset{j=1}{\sum }}∥\tilde{y}(k,j){∥}^{2}+2\overset{\mu }{\underset{j=1}{\sum }}2∥{\hat{\varphi }}_{f,k,j}{∥}^{2}\overset{j-1}{\underset{i=0}{\sum }}\frac{∥\varphi_{f,k-i,j}{∥}^{2}}{∥R_{k-i,j}{∥}^{2}}∥\tilde{y}(k-i,j){∥}^{2} & & \\ & +2\mu ∥{\hat{\varphi }}_{f,k,j}{∥}^{2}\overset{j-1}{\underset{i=0}{\sum }}\frac{∥\varphi_{f,k-i,j}{∥}^{2}}{∥R_{k-i,j}{∥}^{2}}\sigma_{w}^{2}, \end{array}$$ (32)

where $Z_{\mu ,k}=∥{\hat{\varphi }}_{f,k,j}^{T}{\tilde{\theta }}_{k,j}{∥}^{2}$.

By employing the martingale convergence theorem to (32), we have


$$\begin{array}{ccc} E[Z_{\mu ,k}|ℱ(k-1)]\leq & \overset{\mu }{\underset{j=1}{\sum }}2∥\tilde{y}(k,j){∥}^{2}+2\overset{\mu }{\underset{j=1}{\sum }}∥{\hat{\varphi }}_{f,k,j}{∥}^{2}\overset{j-1}{\underset{i=0}{\sum }}\frac{∥{\hat{\varphi }}_{f,k-i,j}{∥}^{2}}{∥R_{k-i,j}{∥}^{2}}∥\tilde{y}(k-i,j){∥}^{2} & \\ & +2\mu ∥{\hat{\varphi }}_{f,k,j}{∥}^{2}\overset{j-1}{\underset{i=0}{\sum }}\frac{∥{\hat{\varphi }}_{f,k-i,j}{∥}^{2}}{∥R_{k-i,j}{∥}^{2}}\sigma_{w}^{2}, \end{array}$$ (33)

By using $R_{k,j}$ to (33), we have


$$\begin{array}{ccc} \frac{E[Z_{\mu ,k}|ℱ(k-1)]}{R_{k,j}}\leq & \overset{k-1}{\underset{i=1}{\sum }}\overset{\mu }{\underset{j=1}{\sum }}\frac{2}{r_{i,j}}∥\tilde{y}(i,j){∥}^{2}+\overset{k-1}{\underset{i=1}{\sum }}\frac{2}{r_{i,j}}\overset{\mu }{\underset{j=1}{\sum }}∥\hat{\varphi }(f,i,j){∥}^{2} & \\ & \times \overset{j-1}{\underset{m=0}{\sum }}\frac{∥{\hat{\varphi }}_{f,k-m,j}{∥}^{2}}{∥R_{k-m,j}{∥}^{2}}∥\tilde{y}(k-m,j){∥}^{2}\\ & +\overset{k-1}{\underset{i=1}{\sum }}\frac{2\mu }{r_{i,j}}∥\hat{\varphi }(f,i,j){∥}^{2}\overset{j-1}{\underset{m=0}{\sum }}\frac{∥{\hat{\varphi }}_{f,k-m,j}{∥}^{2}}{∥R_{k-m,j}{∥}^{2}}\sigma_{w}^{2}\\ = & \frac{2}{R_{k,j}}\overset{k}{\underset{i=1}{\sum }}\overset{\mu }{\underset{j=1}{\sum }}∥\tilde{y}(i,j){∥}^{2}+\frac{2}{R_{k,j}}\overset{k}{\underset{i=2}{\sum }}\overset{\mu }{\underset{j=1}{\sum }}\frac{∥{\hat{\varphi }}_{f,i,j}{∥}^{2}}{R_{i,j}}\\ & \times \frac{\left[R_{i,j-1}-r_{i,0}][∥\tilde{y}(i,j){∥}^{2}+\mu \sigma_{w}^{2}\right]}{R_{i,j}}\\ \leq & \frac{2}{R_{k,j}}\overset{k}{\underset{i=1}{\sum }}\overset{\mu }{\underset{j=1}{\sum }}∥\tilde{y}(i,j){∥}^{2}+\frac{2}{R_{k,j}}\overset{k}{\underset{i=2}{\sum }}\overset{\mu }{\underset{j=1}{\sum }}\frac{∥{\hat{\varphi }}_{f,i,j}{∥}^{2}}{R_{i,j}}\\ & \times [∥\tilde{y}(i,j){∥}^{2}+\mu \sigma_{w}^{2}]. \end{array}$$ (34)

Because of $\sum_{j=1}^{\mu }\sum_{i=1}^{k}\frac{∥{\hat{\varphi }}_{f,i,j}{∥}^{2}}{R_{i,j}}=\leq ln(R_{k,j})$, (34) has the following form:


$$\begin{array}{ccc} \frac{E[Z_{\mu ,k}|ℱ(k-1)]}{R_{k,j}} & \leq \frac{4}{R_{k,j}}\overset{k}{\underset{i=1}{\sum }}\overset{\mu }{\underset{j=1}{\sum }}∥\tilde{y}(i,j){∥}^{2}+\frac{2}{R_{k,j}}\overset{k}{\underset{i=2}{\sum }}\overset{\mu }{\underset{j=1}{\sum }}\frac{∥{\hat{\varphi }}_{f,i,j}{∥}^{2}}{R_{i,j}}(\mu \sigma_{w}^{2}) & \\ & =\frac{4}{R_{k,j}}\overset{k}{\underset{i=1}{\sum }}\overset{\mu }{\underset{j=1}{\sum }}∥\tilde{y}(i,j){∥}^{2}+\frac{2ln(R_{t,j}}{R_{k,j}}(\mu \sigma_{w}^{2})\to 0,as\;k\to \infty . \end{array}$$ (35)

It is a fact that $\lambda_{min}[\sum_{j=1}^{\mu }\sum_{i=1}^{k}{\hat{\varphi }}_{f,i,j}{\hat{\varphi }}_{f,i,j}^{T}]∥{\tilde{\theta }}_{k}{∥}^{2}\leq {\tilde{\theta }}_{k}^{T}[\sum_{j=1}^{\mu }\sum_{i=1}^{k}{\hat{\varphi }}_{f,i,j}{\hat{\varphi }}_{f,i,j}^{T}]{\tilde{\theta }}_{k}\leq \lambda_{max}[\sum_{j=1}^{\mu }\sum_{i=1}^{k}{\hat{\varphi }}_{f,i,j}{\hat{\varphi }}_{f,i,j}^{T}]∥{\tilde{\theta }}_{k}{∥}^{2}$, it has


$$\begin{array}{ccc} \begin{array}{ccc} \frac{\lambda_{min}[\overset{\mu }{\underset{j=1}{\sum }}\overset{k}{\underset{i=1}{\sum }}{\hat{\varphi }}_{f,i,j}{\hat{\varphi }}_{f,i,j}^{T}]∥{\tilde{\theta }}_{k}{∥}^{2}}{R_{i,j}} & \leq \frac{{\tilde{\theta }}_{k}^{T}[\overset{\mu }{\underset{j=1}{\sum }}\overset{k}{\underset{i=1}{\sum }}{\hat{\varphi }}_{f,i,j}{\hat{\varphi }}_{f,i,j}^{T}]{\tilde{\theta }}_{k}}{R_{i,j}} & \\ & =\frac{E[Z_{\mu ,k}|ℱ(k-1)]}{R_{k,j}}\to 0,\;k\to \infty . \end{array} & & \end{array}$$ (36)

Because of $R_{k}=O(\lambda_{min}[\overset{k}{\underset{m=1}{\sum }}\overset{\mu }{\underset{n=1}{\sum }}{\hat{\varphi }}_{f,m,n}{\hat{\varphi }}_{f,m,n}^{T}]),k\to \infty$, the above equation can be written as follows:


$$\begin{array}{ccc} ∥{\tilde{\theta }}_{k}{∥}^{2}=O(R_{k}∕(\lambda_{min}[\overset{k}{\underset{i=1}{\sum }}\overset{\mu }{\underset{j=1}{\sum }}{\hat{\varphi }}_{f,i,j}{\hat{\varphi }}_{f,i,j}^{T}]))\to 0,a.s.k\to \infty . & & \end{array}$$ (37)

To this end, the theorem 1 has been completed. □

A conclusion can be drawn from Theorem 1 that the parameter estimation error can converge to zero by using the proposed method for the considered nonlinear sandwich system.

## 5 Case study

### 5.1 Simulation example

In this section, we introduce simulation case to test and verify the results proposed in this paper. In the simulation, we take into account the data filtering based multi-innovation gradient method (D-MISG) [42] and Three-step algorithm [15] as comparison algorithms.

**Fig 3:**
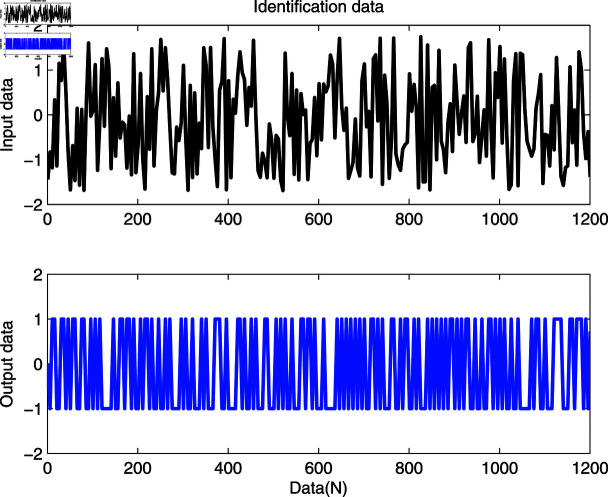
Identification data.

The dynamics of the Sandwich systems subject to friction nonlinearity and quantizer sensor are described by:


$$\begin{array}{ccc} x_{k}=\frac{u_{k-1}+0.2u_{k-2}}{1+0.55x_{k-1}+0.35x_{k-2}}, & & \end{array}$$ (38)

$$\begin{array}{ccc} \begin{array}{c} v_{k}=0.01x_{k}-0.01x_{k-1}+0.02sign(x_{k}-x_{k-1})-0.5\tanh(x_{k}-x_{k-1}), \end{array} & & \end{array}$$ (39)

$$\begin{array}{ccc} y_{k}=\frac{v_{k-1}+1.1v_{k-2}}{1+0.8x_{k-1}+0.6x_{k-2}}+\frac{w_{k}}{1+0.8x_{k-1}+0.6x_{k-2}}, & & \end{array}$$ (40)

$$\begin{array}{ccc} y_{q,k}=C(y_{k}), & & \end{array}$$ (41)

**Fig 4:**
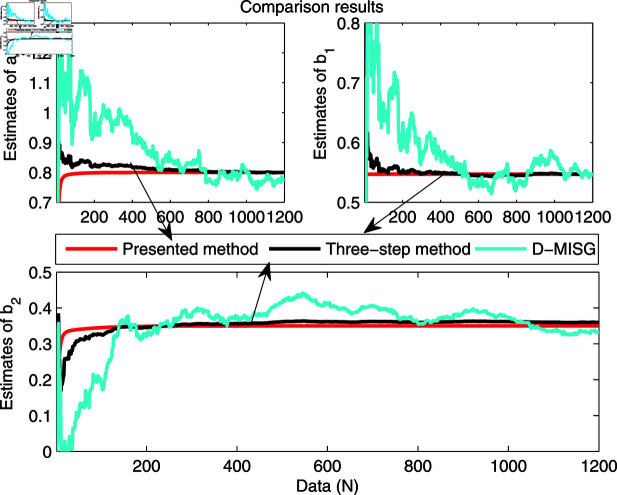
Estimates of $L_{1}$.

**Fig 5:**
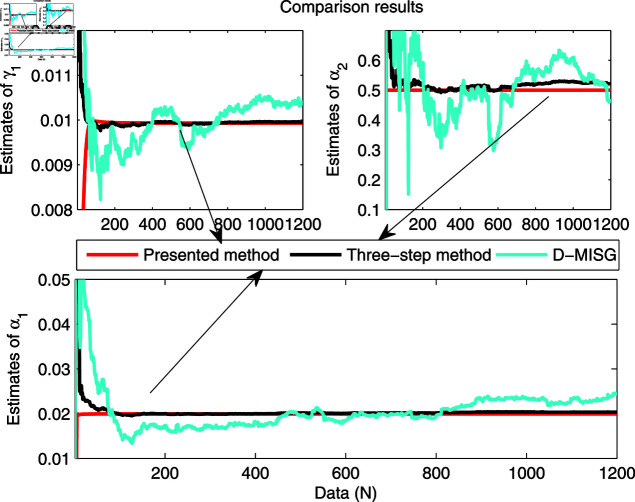
Estimates of friction model.

**Fig 6:**
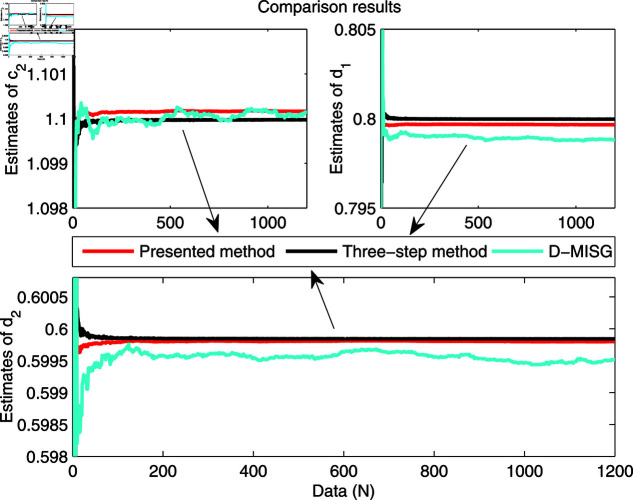
Estimates of $L_{2}$.

The identification data are collected, as displayed in Fig 3, which is conducted by using a random sequence *N* ∈ ( 0 , 1 )  and an additional white noise *N* ∈ ( 0 , 0 . 01 ) . The initial parameters of the developed method are listed as: $\eta_{0}=1.5$, *ξ* = 0 . 96, *μ* = 4, ${\hat{\theta }}_{0,0}=I∕p0$, $p0=10^{3}$, $R_{0,0}=1$, $x_{ref,0}=I∕p0$, and $v_{ref,0}=I∕p0$. From the results plotted in Fig 3, compared with the features of the input signal,the quantized output data contain limited system information, which makes quantized identification work difficult. The following parameter estimation from results that are presented in Figs 4, 5 and 6. One can get that the faster and higher convergence performance are reflected by estimation results about the proposed method than the D-MISG and three-step methods. More specifically, the D-MISG method cannot converge to its expect values because of the problem of noise data utilization. The Three-step method has better performance than the D-MISG thanks to the multi-stage principle.

**Fig 7:**
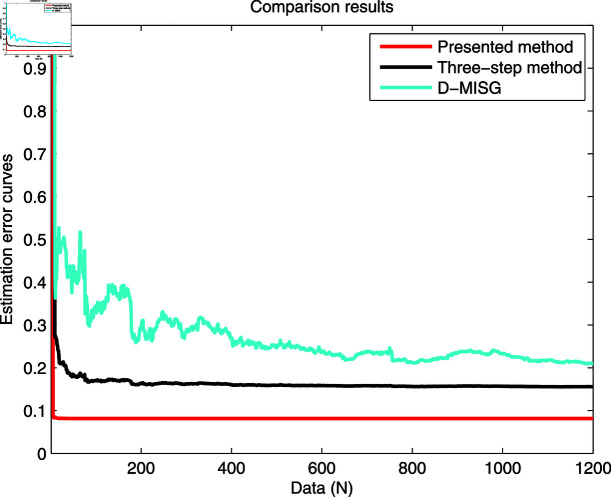
Parameter estimation errors.

**Fig 8:**
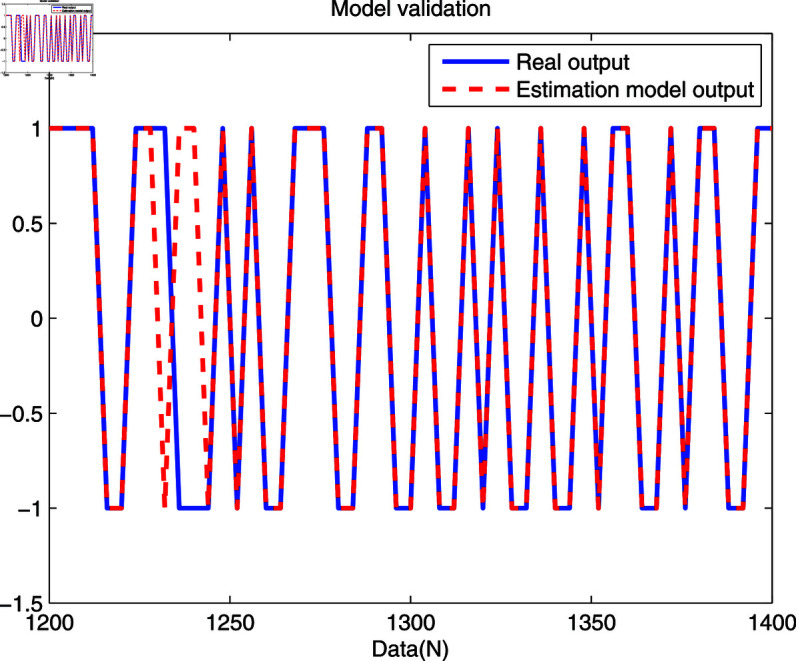
Comparison model output.

Fig 7 illustrates the estimation error histories with the three estimators along with the increase of amount of samples. From this figure, it can obviously be seen that the estimation errors by three estimators are rapidly converging at the initial stage of the estimators, and subsequently approach the small values at the final trial. The above-phenomenon indicates that the three algorithms can estimate the parameters of the studied system with different properties. More thorough-going analysis from Fig 7 demonstrates that the proposed algorithm takes less time to find the expected values and has a smaller steady-state error than the other two identifiers. Overall, the parameter estimation results shown in Figs 4, 5, and 6 are highly consistent with the estimation error curves displayed in Fig 7.

**Fig 9:**
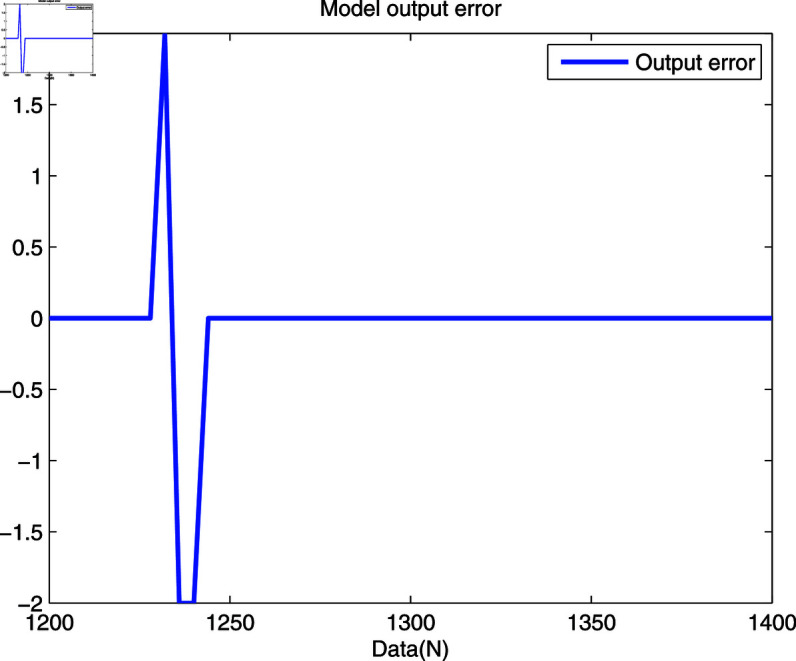
Model error.

In Figs 8 and 9, the real trajectory and the model output trajectory of the developed method are shown. One can see that the error between the actual output and model output are small at most sampling points, while some error data are abnormally large. It is because quantized model output cannot effectively track the actual output, which is determined through the unique features of the quantized sensor.

**Fig 10:**
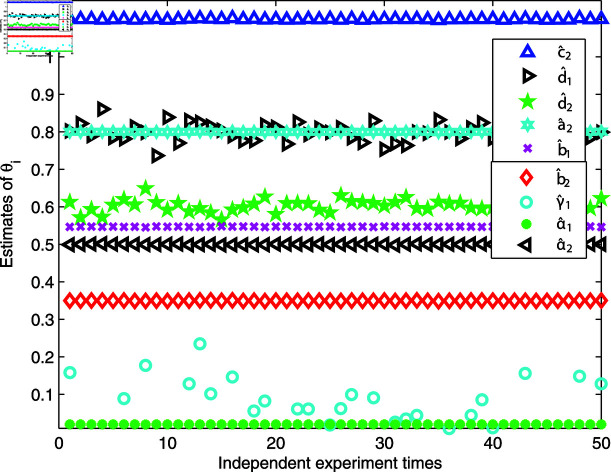
Parameter distributions.

**Fig 11:**
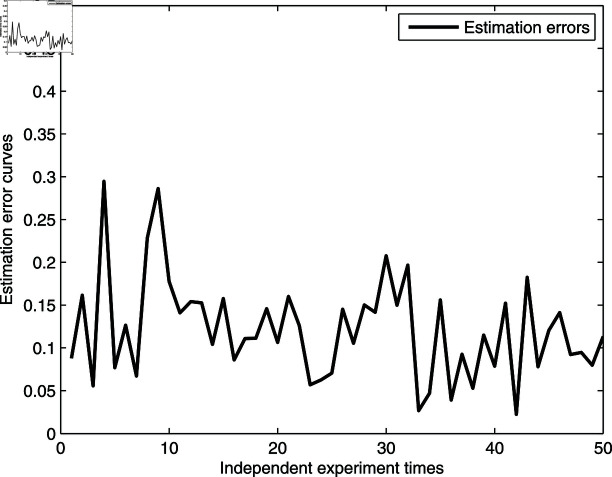
Estimation error distributions.

Figs 10 and 11 provide illustrations of the parameter distribution and changes in estimation error based on 50 independent experiments. It is clear that most estimated parameters are concentrated around the corresponding expected values, except for ${\hat{\gamma }}_{1}$. From Fig 10, we also find that the parameter distribution of the first linear dynamic $L_{1}$ are more compact than the friction model and the second linear system $L_{2}$, and the reason is that the friction model and the second linear system are at the back end of the research system and are more prone to signal distortion when a quantizer is used. Although the parameter estimation errors in Fig 11 vary greatly, the estimation error values remain relatively small, and the estimation errors are uniformly distributed in the middle and later stages of experimental testing, indicating that the estimator gives a strong stability and is effective.

**Fig 12:**
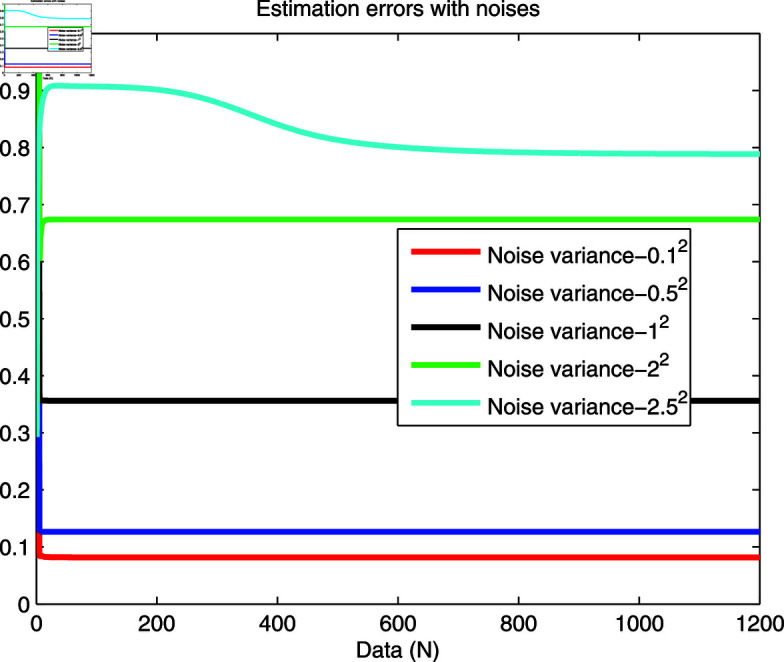
Estimation performance with different intensities.

Fig 12 is used to show the estimation performance against noise intensities. It is clearly observed that the decrease in estimation performance caused by an appropriate increase in noise intensity (e.g., 0.1^2^, 0.5^2^ and 1^2^) is acceptable, thereby indicating the robustness of the proposed estimator. When stronger noise is used, the estimation performance is poor (e.g, 2^2^). It may even be unacceptable (e.g, 2.5^2^) because the useful data of the system are overwhelmed, and noise dominates completely. A comparison of the existing reports [23,27,29], it has been found that when the noise density increases to 2^2^, the performance of the estimator is already poor compared with the method of this paper, thus demonstrating the effectiveness of the presented method.

### 5.2 Real plant

**Fig 13:**
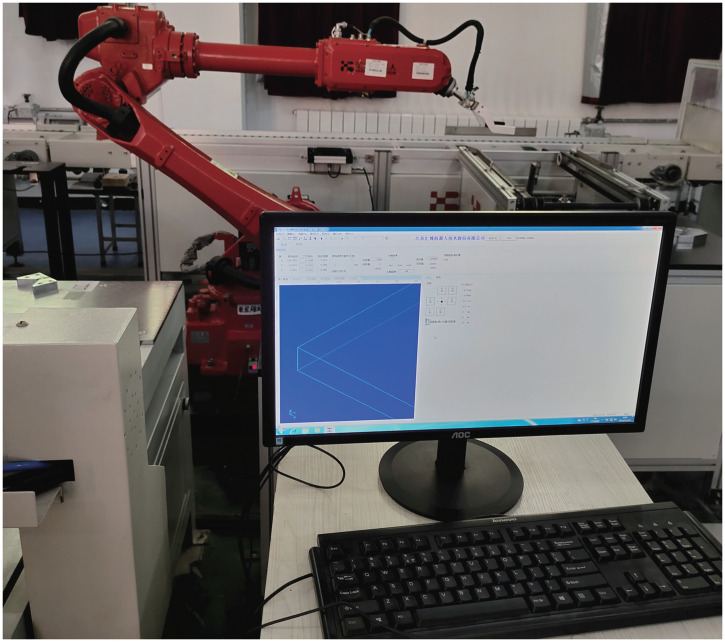
Electromechanical system.

In the experiment, we have selected a given signal $y_{d}=sin(3∕5\pi t)$ to excite the electromechanical system with a binary sensor, as plotted in Fig 13, to obtain the identification data. The test-bench consists of a drive motor (HF-SP702BK) with a digital signal processor (TMS320F28335), an encoder (OIH60-TS5246 N473) and a code composer studio (CCS) v4 for data visualizing with a PC. The dynamic description of the electromechanical system is written as [60,61]

**Fig 14:**
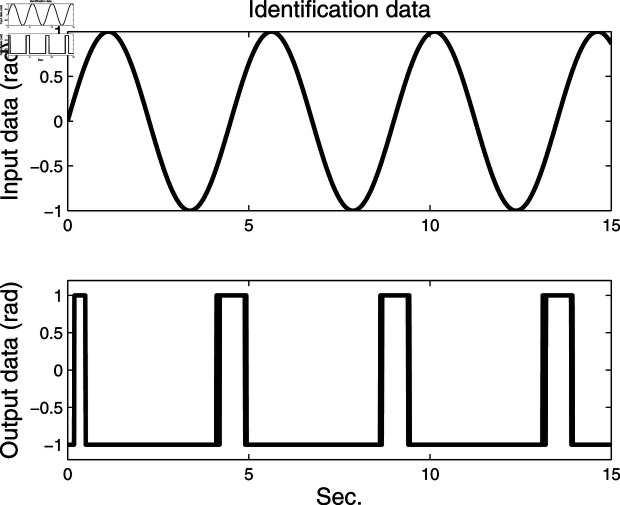
Input and output data of system.

**Fig 15:**
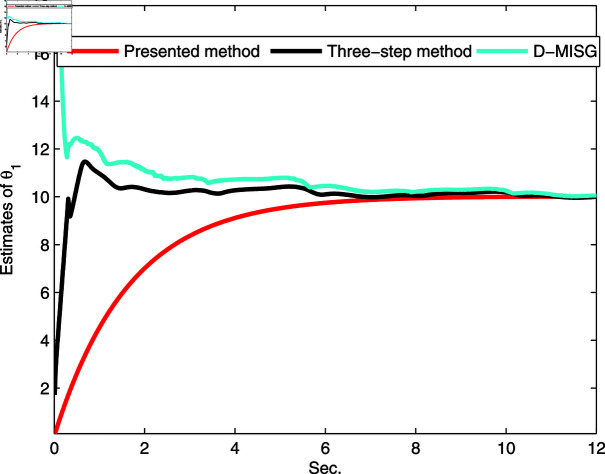
Comparison estimation of $\theta_{1}$.

**Fig 16:**
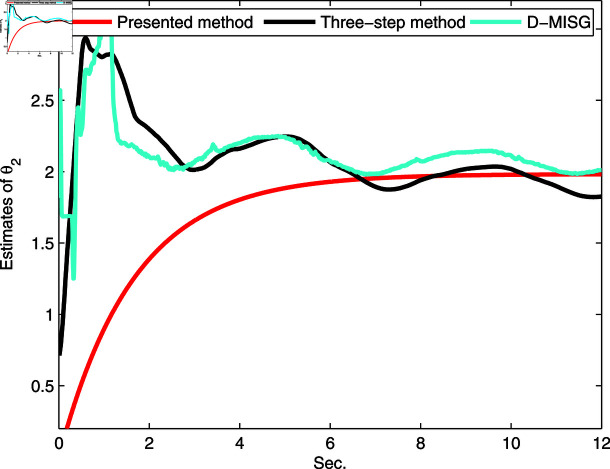
Comparison estimation of $\theta_{2}$.

**Fig 17:**
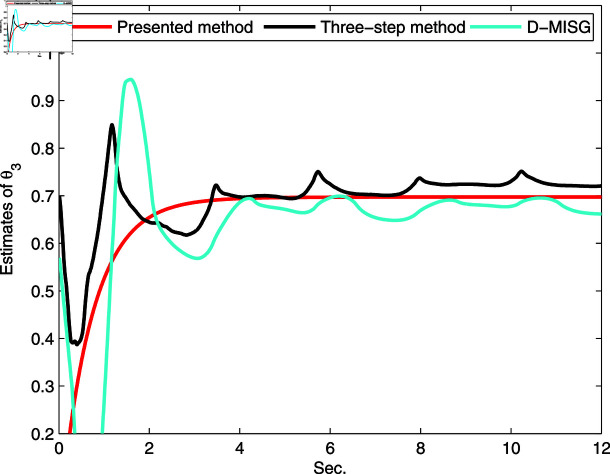
Comparison estimation of $\theta_{3}$.

**Fig 18:**
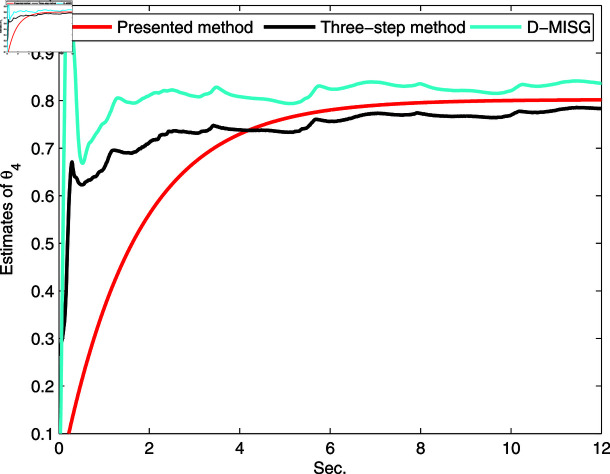
Comparison estimation of $\theta_{4}$.


$$\begin{array}{ccc} \{\begin{array}{c} {ẋ}_{1}=x_{2}\\ {ẋ}_{2}=\frac{1}{J_{L}}(-C_{2}x_{2}+C_{1}u-T_{c}sign(x_{2})-bx_{2}-T_{f}) \end{array} & & \end{array}$$ (42)

where $J_{L}$ is inertia of motor, which is the ability of the motor to resist speed changes during rotation, and it can reflect the acceleration ability and torque performance of the motor. $C_{2}=K_{c}K_{d}∕R_{g}$ and $C_{1}=K_{c}∕R_{g}$. $R_{g}$ denotes the resistance of armature, which is the resistance of the armature coil in the motor, and it is inversely proportional to the magnitude of the torque. $K_{c}$ represents electromechanical coefficient, which expresses that the degree of the conversion of mechanical energy into electrical energy. $K_{d}$ denotes back-electromotive constant, which generates an electromotive force due to changes in the magnetic field, reflecting the electromagnetic torque of the motor. *T_c_* is a coulomb friction coefficient, and *b* is coefficient of viscous friction [62]. $T_{f}$ denotes the load.

The estimated vector $\theta ={\left[C_{2}∕J_{L},C1∕J_{L},T_{c}∕J_{L},b∕J_{L}\right]}^{T}=\theta_{i},i=1,2,3,4$. Figs 15–18 display the parameter estimation vector $\theta_{i}$ for the proposed method, D-MISG, and three-step approaches based on the identification data shown in Fig 14. The estimation performance of the presented method outperforms the D-MISG and three-step approaches by around 8 seconds. Moreover, the proposed method provides faster convergence performance than the other two estimators. Although the D-MISG has convergence and accuracy properties that are comparable with the three-step approach, it has significant oscillations. Compared with the presented scheme, the D-MISG and three-step methods have roughness estimation curves, thus demonstrating the contributions of the internal iteration principle and the data filtering technique.

**Fig 19:**
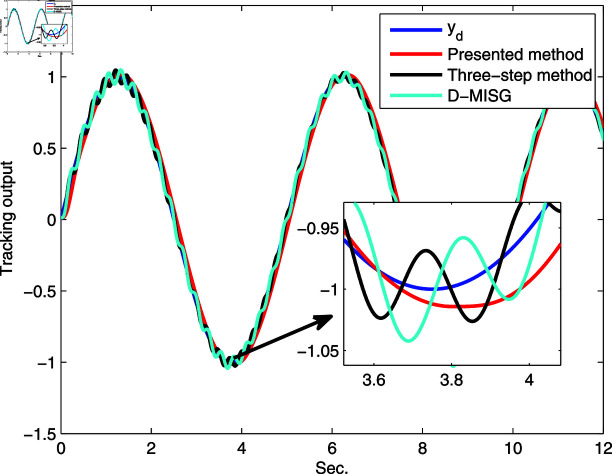
Comparison tracking.

**Fig 20:**
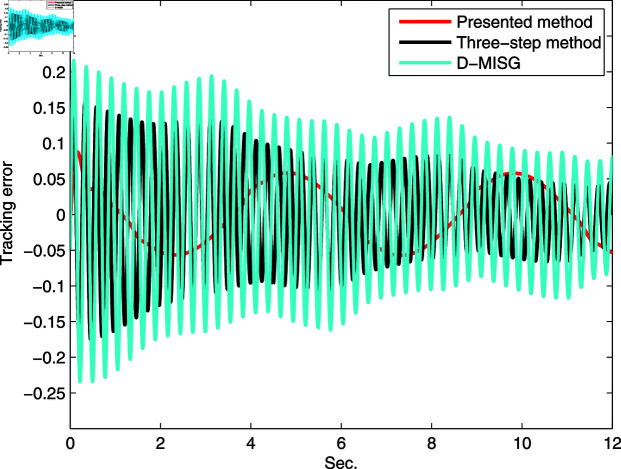
Tracking error.

In Figs 19 and 20, we plot the tracking output and error based on the estimation results of the three estimators. It can be observed that the presented internal iteration scalar-innovation gradient method and the other two estimators are able to track the changes in the sine signal response of the real system, thus indicating the effectiveness of the established estimation models. All tracking errors are small and acceptable, and the proposed estimator has smallest error than the D-MISG and three-step approaches. It can be concluded from Figs 19 and 20 that the proposed method has high-performance and can restore the parameter data of the considered real system compared with the other two methods, and thus achieves the smallest system misadjustment performance, suggesting the advantages and usefulness of the developed method in this work.

## 6 Conclusion

In this study, the performance of the classical MISG method has been improved by merging a new estimator based on the internal iteration principle and the data filtering technique. The priority target was to identify the parameter information of the studied system by using the proposed method. The developed algorithm introduced the internal iteration principle to revise the utilization of multi-innovation participation in parameter adaptive updates and enhance the estimation performance. The presented adaptive filter used the system dynamic to adjust the data preprocessing performance and reduce the bias estimation of the real parameters of the system. The triggering mechanism is used to improve the convergence rate of the estimator. Simulation results, including the parameter estimation results, estimation error curves, independent testing experiment, noise resistance test, and model validation, indicate that the proposed algorithm has exceptional estimation performance compared with several existing estimation methods. The actual experimental results of the proposed algorithm on electromechanical systems have also demonstrated the advantages and usefulness of the designed algorithm.

As a new exploration in quantized system estimation, this report starts with fundamental yet straightforward system settings. Future research augment the current study by focusing on multithreshold quantizers, multivariable systems, and network attack systems.

## Supporting information

S1 Text Experimental raw data. (XLSX)
